# Clinical evaluation of a modified silver fluoride application technique designed to facilitate lesion assessment in outreach programs

**DOI:** 10.1186/1472-6831-13-73

**Published:** 2013-12-28

**Authors:** Graham G Craig, Keith R Powell, Carole A Price

**Affiliations:** 1 Dental Outlook, PO Box 275, Camperdown, NSW 1450, Australia; 2 Private practice, 34 Church Street, Burwood, NSW 2134, Australia

**Keywords:** Silver fluoride, Clinical trial, Carious lesion assessment

## Abstract

**Background:**

An advantage of using silver fluoride treatments for carious primary teeth in outreach programs especially where dental resources are limited is that the treatments can be carried out by dental auxiliaries. One limitation to date is that assessments of lesion status have been based on a tactile test where a sharp probe or explorer is drawn across the surface of a lesion to assess its hardness. This is a technique-sensitive step and has the potential for iatrogenic damage, especially when a lesion is deep. This study was undertaken to determine whether an alternative, non-invasive, visual assessment could be a reliable indicator of lesion status. The approach was based on the retention, or otherwise, of a black surface deliberately created at the time of initial treatment.

**Methods:**

A total of 88 lesions in the primary molars of 45 children, aged 5 to 10 years, were treated with a one-minute application of 40% silver fluoride. The surface of the lesions was then deliberately turned black by the application of 10% stannous fluoride as a reducing agent. All lesions were on an approximal or occlusal surface of a first or second primary molar. The presence or absence of a continuous black surface at 6 months and any changes in radiographic depth that had occurred in that period were determined from digitized photographs and bitewing radiographs.

**Results:**

The retention of an uninterrupted black surface was associated with minimal or no caries progression whereas lesions with an incomplete or lost black surface were 4.6 times more likely to have progressed. Use of the Datta and Satten Rank-Sum Test to account for any clustering effect showed that the difference was statistically significant (p < 0.0001). The sensitivity and specificity of the approach were 80% and 81% respectively.

**Conclusion:**

The retention of a continuous black surface after the application of silver fluoride followed by a reducing agent on carious lesions in primary molars can provide a useful visual indicator of lesion progression and so be relevant for use in dental outreach programs.

## Background

One of the current problems in many areas of the world is that the necessary restorative treatments are not available due to poor access to dental care and limited financial resources [1]. Recent reviews have highlighted the potential of silver diammine fluoride as one of the approaches to help this situation with child patients [2,3]. The topical application of 38% silver diammine fluoride has been found to have a significant effect on arresting caries progression in primary teeth [4-7]. A similar result was obtained with lesions in first permanent molar teeth [5]. A water-based 40% silver fluoride has also been used to treat open lesions in primary teeth [8]. Another study found it to reduce pit and fissure lesions in newly erupted first permanent molar teeth [9].

The nature of the silver fluoride application procedure facilitates its use by dental auxiliaries. However, a limitation to date is that the status of treated lesions has been based on a tactile assessment where an explorer or sharp probe is used to determine the hardness of the lesion surface [4-7]. This method is technique sensitive and care is needed not to cause iatrogenic damage, especially if the lesion is deep [7]. Although a black surface is a characteristic of arrested silver fluoride-treated lesions, color change in itself has not been used to assess lesion status [4-7]. However, a previous empirical observation suggested that color change alone may a useful indicator of lesion progression if the surface of the lesion is deliberately turned black shortly after the silver fluoride application [8]. A reducing agent, stannous fluoride, was used for this purpose. If the black surface remained it was suggestive of lesion stasis and, if it was lost, it provided a possible indication of lesion progression.

This study was undertaken to determine whether, by using a reducing agent and deliberately turning the surface of a lesion black after a silver fluoride application, subsequent color changes could provide a useful indicator of lesion status.

The present paper reports on the changes in radiographic depth and surface color of open occlusal- and approximal –surface lesions in primary molars after the topical application of 40% silver fluoride followed by the use of stannous fluoride as a reducing agent.

## Methods

The study was carried out on children living in Bourke, an isolated town in western New South Wales, Australia. The children had a high decay rate and dental health resources were limited in the low socio-economic community. The protocol was approved by the Principal Dental Officer (Orana and Far Western Region) and ethical approval obtained. A letter was sent to the children’s parents explaining the purpose of the trial and the procedures to be followed. Written consent was received from the parents of all the children.

The initial visit involved patient examination and the taking of radiographic and photographic records. Posterior bitewing radiographs were taken using a standardized technique with a 56 kV X-ray machine (Siemens Heliodent) and fast dental X-ray film (Kodak Periapical Ultra Speed DF 54) and developed using a standardized, automated procedure. Before assessment the radiographs were digitized using a film scanner (OpticFilm 7600i Film Scanner, Plustek, Santa Fe Springs, USA) and then stored as grey-scale images in a high-resolution JPEG format. Photographs of the occlusal surfaces of primary molars were taken at 1.5 × magnification (Nikon camera fitted with a 200 mm Medical Nikkor lens, Nippon Kogahu K.K., Tokyo). The film was subsequently developed in a film laboratory using a standardized technique. Before assessment the photographs were digitized using a film scanner (OpticFilm 7600i Film Scanner, Plustek, Santa Fe Springs, USA) and then stored in a high-resolution JPEG format. The parameters of the scanning process were adjusted to ensure that the spectral composition of each digitized photograph was similar.

All the open carious lesions selected for the study were active and had extensive dentine involvement. All lesions were initiated on either the approximal or occlusal surfaces of primary molars. The approximal surface lesions had reached a stage of progression where there was breakdown of the overlying marginal ridge. All walls and the floor of each lesion had to be clearly visible at baseline. These parameters were necessary for lesion progression to be assessed from bitewing radiographs and for the presence or absence of an uninterrupted surface film to be assessed from photographs.

Lesions, without an accompanying pulp exposure, that met these requirements were treated with a solution of 40% silver fluoride (Creighton Pharmaceuticals, Sydney) for 60 seconds. This was immediately followed by the application of a 10% stannous fluoride paste (Creighton Pharmaceuticals, Sydney). A piece of dissolving adhesive wafer (Stomahesive Wafer, Squibb and Sons, New York) was adapted over each treatment site and then allowed to dissolve in the oral fluids. Clinical examination showed that all treated lesions had an uninterrupted black surface 24 hours after treatment.

At the 6-month recall visit a clinical examination was carried out and bite-wing radiographs and photographs taken as described previously.

For assessment, the digitized radiographic images were opened in photo editing software (Adobe Photoshop CS2, version 9.2.11) and each large image cropped into one or more smaller images so that there was only one treated tooth per image. To compensate for any minor variations in projection angles during taking of the radiographs at baseline and 6 months, geometric alignment of the images was carried out as described previously [10]. This was done using image registration software (Regeemy Software, Image Registration and Mosaicking,v.2.43: Instituto National de Pesquisas, Sao Jose dos Campos, SP, Brazil). Following alignment, each baseline and follow-up image was placed into graphic design software (Adobe Illustrator CS2, version 9.2.11) on a black background and magnified 4.275 times. The image pairs were alternated at random so that the subsequent examiner was blinded as to which was a baseline or follow-up image. For assessment of lesion depth, the subsequent examiner marked the base of the lesion and the corresponding pulp section using the brush tool at a fine setting. The resulting image was printed out and the caries-pulp distances measured. A second series of measurements were made one week later.

Digitized photographs of the selected lesions taken at 6 months were inspected by two examiners working independently. Each photograph was opened in photo editing software (Adobe Photoshop CS2, version 9.2.11) and viewed under standardized lighting conditions. Lesions were classified on one criterion only: whether they possessed an uninterrupted black surface or not.

For data analysis, as a preliminary step, the first and second radiographic readings of lesion depth at baseline and the two readings at 6 months were examined to determine the validity of using the mean values for subsequent statistical analyses. The Kolmogorov-Smirnov Test was used to assess the normality of the distribution of the differences between the first and second readings. The median differences were compared employing the Wilcoxon Signed Rank Test. The Pearson Product Moment Correlation Coefficient and the Spearman Rank Correlation Coefficient were calculated to give correlations between the two measurements at baseline and the two at 6 months. The Kappa Index of Agreement was used to measure intra-examiner agreements between duplicate readings of photographs. These statistical tests were performed using the appropriate statistical package (SAS Version 9.2, SAS Institute Inc, Cary, USA). Because some subjects contributed multiple observations to the dataset, including observations to both experimental groupings, the observations were clustered at the subject level. Therefore a non-parametric, modified Wilcoxon Rank-Sum Test as proposed by Datta and Satten was employed [11]. The test was performed utilizing open access software (R Version 2.13.2 http://cran.rproject.org/).

The adequacy of the sample size was checked once the readings were taken to ensure a 5% statistical significance level and an 80% power.

## Results

A total of 88 lesions in 45 subjects that met the inclusion criteria were available for examination at 6 months. The cohort comprised 25 males and 20 females of mean age 7.4 ± 1.2 years at baseline. The mean number of decayed, missing and filled primary tooth surfaces (dmfs) in the cohort at baseline was 12.6 ± 6.0.

The preliminary statistical analysis of the two measurements of each lesion depth at baseline and at 6 months showed that the distribution of the differences between readings one and two at each time interval revealed non-normality (Kolmogorov-Smirnov p-value <0.01). However, the Wilcoxon Signed Rank Test showed the median differences were not significantly different from zero. The correlations between the two measurements of radiographs taken at baseline were high. The Pearson product moment correlation coefficient was 0.9545 (p-value < 0.0001, H_0_: ρ = 0), whilst the Spearman rank correlation coefficient was 0.9162 (p-value < 0.0001, H_0_: ρ = 0). The correlations between the two measurements of radiographs taken at 6 months were also high. The Pearson product moment correlation coefficient was 0.97161 (p-value < 0.0001, H_0_: ρ = 0), whilst the Spearman rank correlation coefficient was 0.9457 (p-value < 0.0001, H_0_: ρ = 0).

There was sufficient repeatability in these depth measurements to use the mean of the two readings for each lesion at both time intervals for the subsequent analyses.

Presented in Table 1 is the summary of changes in lesion depth by surface type observed at 6 months. A total of 47 lesions demonstrated an uninterrupted black surface whilst 41 either had an interrupted or a lost black surface. The median increase in caries depth for lesions retaining a black surface was −0.03 mm (S.D. = 0.23) compared to 0.24 mm (S.D. = 0.25) for lesions with an interrupted/lost black surface. The Datta and Satten Rank-Sum Test found that the distributions of the two groups was highly significant giving a test statistic of 8.05 and a two-sided p-value <0.0001. The probability values confirmed that the sample size was quite adequate.

**Table 1:** Summary of changes in lesion depth by surface type over 6 months

**Change in caries-pulp distance at 6 months (mm)**							
**Lesion surface type**	**n**	**Mean**	**Median**	**Std.dev.**	**Min.**	**Max.**	**Inter-quartile range**
Uninterrupted black	47	0.00	−0.03	0.23	−0.59	0.83	−0.12,0.00
Non-retained/interrupted black	41	0.26	0.24	0.25	−0.46	0.70	0.12,0.47

Table 2 shows the distribution of lesions with and without an uninterrupted black surface by site of initiation and median change in lesion depth over 6 months. In terms of site of initiation, 25% of the lesions originated on the occlusal surface and 75% on the approximal surfaces of the primary molars. All the approximal surface lesions occurred on either the distal surface of the first primary molars or the mesial surface of the second primary molars. At these sites the median change in depth for lesions with an uninterrupted black surface was less than that for lesions with an interrupted or lost black surface. The Datta and Satten Rank-Sum Test found that the distributions of changes in lesion depth at these sites all reached significance at the 5% level.

**Table 2:** Median change in lesion depth over 6 months by caries initiation site

**Site of lesion Initiation (surface)**	**n**	**Uninterrupted**			**Interrupted or lost**			**Datta and Satten two-sided p-value**
		**Black surface**			**Black surface**			
		**Median (mm)**	**n**	**IQR**	**Median (mm)**	**n**	**IQR**	
Occlusal	22	−0.05	13	−0.22, 0.0	0.41	9	0.24, 0.59	0.0131
Approximal	66	−0.03	34	−0.09, 0.0	0.24	32	0.09, 0.41	0.0003
Total	88	−0.03	47	−0.12,0.00	0.24	41	0.12,0.47	<0.0001

A determination of the inter-examiner agreement for photographic assessments gave a Kappa coefficient of 0.9.

Data used in a probability analysis are given in Table 3. The proportion of lesions with an interrupted or lost black surface that had decay progression was 0.78 (32/41). In comparison, the proportion of lesions with an uninterrupted black surface that had decay progression was 0.17 (8/47). The relative risk of decay progressing for lesions with an interrupted or lost black surface (compared to those with an uninterrupted black surface) was 0.78/0.17 = 4.6 indicating that these lesions had a 4.6 times greater likelihood of progressing during the study period.

**Table 3:** Probability analysis of lesion progression

**Lesion surface colour**	**Decay did not progress n (% of row total)**	**Decay progressed n (% of row total)**	**Total**
Interrupted/lost black	9 (22%)	32 (78%)	41
Uninterrupted black	39 (83%)	8 (17%)	47
Total	48 (55%)	40 (45%)	88

Of the 40 lesions that progressed during the study 32 had an absent or disrupted black surface giving a sensitivity of 80% and, of the 48 lesions that did not progress, 39 had an uninterrupted black surface giving a specificity of 81%.

## Discussion

All lesions in this study were open and had extensive dentine involvement. Whether or not the treatment with silver fluoride followed by stannous fluoride had an effect in slowing or arresting these lesions is not known, as the investigation was not designed to evaluate this aspect. However, the possibility does exist as recent reviews have attested to the efficacy of silver fluoride preparations, in particular 38% silver diammine fluoride, in arresting carious lesions in primary teeth [2,3]. The mode of action has been attributed to an inhibition of the demineralization process and a protective effect against collagen degradation [12]. In addition, 38% silver diammine fluoride has demonstrated anti-microbial activity against organisms in an in vitro biofilm prepared using multiple cariogenic bacteria [13].

The retention of an uninterrupted black surface on open carious lesions 6 months after the application of silver fluoride followed by stannous fluoride was associated with a statistically significant reduction in lesion progression. It is noteworthy that this effect was seen at the major sites of caries initiation in primary molars and it occurred even though the cohort had a high caries rate as shown by the elevated mean decayed, missing and filled surfaces score at baseline. The usefulness of the black surface color as a predictor of caries activity was shown by the finding that lesions with an interrupted or lost black surface were 4.6 times more likely to have decay progression. Also the sensitivity of the approach in the context that an incomplete or absent black surface was a diagnostic aid for decay progression was 80%, whereas the specificity referring to situations where an uninterrupted black surface was present and decay had not progressed was 81%.

Several studies have reported the black staining of arrested carious dentine lesions in primary teeth at some stage after the application of 38% silver diammine fluoride [4-7]. How soon after the application these changes occurred is not known because, in all these studies, 6 months elapsed before the treated lesions were re-examined. An exception was one of the three treatment groups in one of the studies [6]. In this group the tannic acid from tea was used as a reducing agent to turn the surface of a lesion dark brown after the silver diammine fluoride application. However, this color change was not used to monitor lesion progression. When a deliberate reducing agent is not used, the rate of development of any color change would depend, amongst possible other factors, on the amount of reducing agents already in the lesion. However, if color is being used as an indicator of lesion progression, this variable is eliminated and a standardized baseline color is obtained by the use of a reducing agent as the second stage of a silver fluoride treatment.

The data in this study were clustered and, because of this, the Datta and Satten Rank Sum Test was used for statistical analysis rather than the generalized estimating equation (GEE) approach that generalizes the T-test [14]. The GEE approach is more sensitive to outliers than non-parametric, rank-based approaches and both groupings in this study had outliers. Because it was desired to compare lesions with and without an interrupted black surface at specific tooth sites, the relatively small number of lesions would have limited the ability to correctly specify a GEE working correlation structure.

The composition of the surface stain and why its loss or break up is associated with lesion progression have not been established. However, it is possible that the underlying lesion becomes softer and more hydrated resulting in a loss of support and a breakup of the surface deposit.

Use of a silver salt solution followed by a reducing agent is a standard technique for the argyrophil staining reaction in histology [15]. In such an application free silver ions bind with various functional groups in the tissue and are reduced to metallic silver. These become catalytic sites for further silver deposition once the reducing agent is applied and a large number of silver ions are reduced (Ag^+^ > Ag^o^) [15]. Some silver oxide is probably also present [15]. Extrapolating this to the present study, the stannous ions from the stannous fluoride acted as electron donors to reduce the free silver ions to metallic silver. The end result was probably a deposit of which the mineral portion comprised mainly silver and silver oxide.

In this study, the distinctive black surface color led to a high degree of reproducibility when lesions were examined in photographs taken 6 months after the initial treatment (Kappa coefficient = 0.9). Also the clear caries outline in many radiographs facilitated the high degree of reproducibility of duplicate lesion-depth measurements as evidenced by the high Pearson product moment correlation coefficient and Spearman rank correlation coefficient. With the digitized radiographs, adjustments for minor differences in projection angles between radiographs taken at baseline and 6 months were made using Regeemy Software. When using this approach it was found necessary, after placing the corresponding reference points (tie points) on each radiograph, to double check this aspect before producing the corrected image. For some reason the program produced a number of completely unrelated tie points that had to be removed.

Assessments of lesion progression from bitewing radiographs showed that, with some lesions, the distance between the lesion and the pulp actually increased in the 6-month observation period. This occurred even though the lesions were open. A similar phenomenon of an increase in the cavity-pulpal distance with time has been reported previously [16]. It was found that, when lesions in primary molars were first treated with silver fluoride followed by stannous fluoride and later restored with resin composite, in 37% of the lesions the distance between the base of the lesion and the pulp increased by 0.2 mm or more over 18 months. Presumably this was due to the deposition of reactionary (tertiary) dentine.

A limitation of the approach used in this study was that the degree of openness of a lesion was not taken into account and related to the retention or otherwise of the black-mat surface. This aspect is worthy of future investigation as it has long been known that open carious lesions in primary teeth progress less rapidly than closed ones [17].

The current findings show that the presence of an uninterrupted black surface on open carious lesions in primary molars is a good indicator of caries stasis following the application of silver fluoride then stannous fluoride. In this study it applied even though the children had a high caries rate. A potential advantage of the approach in outreach programs where silver fluoride applications are being applied and assessed by dental auxiliaries is that it should facilitate easy recognition of lesion changes. By not requiring the use of explorers or probes to assess the hardness of a lesion surface it should also reduce the possibility of iatrogenic damage especially when a lesion is deep.

## Conclusions

Based on the results of this study, the null hypothesis is rejected. It is concluded that the retention of a black surface on an open carious lesion following treatment with silver fluoride followed by stannous fluoride was a reliable visual indicator of lesion stasis.

## Competing interests

The authors declare that they have no competing interests.

## Authors’ contributions

GC participated in the study’s design, carried out the treatments and took photographs in the original study, provided one set of readings for digitized photographs and drafted the current paper. KP participated in the study’s design, carried out treatments, took the radiographs in the original study. CP was the qualified independent examiner who carried out the readings on the digitized radiographs and photographs. All authors read and approved the final manuscript.

## Pre-publication history

The pre-publication history for this paper can be accessed here:

http://www.biomedcentral.com/1472-6831/13/73/prepub
